# Supplementary material for: Single-photon microscopy to study biomolecular condensates
Source: Nat Commun. 2023 Dec 12;14:8224. doi: 10.1038/s41467-023-43969-7 (PMC10716487; doi:10.1038/s41467-023-43969-7)
Supplement: Supplementary file 1 — Supplementary Information [file 41467_2023_43969_MOESM1_ESM.pdf]

SUPPLEMENTARY INFORMATION
Single-photon microscopy to study biomolecular condensates

Supplementary Note 1 - FCS theory

Fluorescence Correlation Spectroscopy (FCS) is a statistical correlation method based on the detected single-molecule fluctuation of fluorescence intensity which can be used to describe the movement of fluorescently tagged molecules. It was the first method, within the bigger family of Fluorescence Fluctuation Spectroscopy (FFS) to be experimental and theoretical developed, and it represents the baseline method for all the subsequent implementations. Here, there will be a brief step-wise mathematical derivation of the autocorrelation function (ACF). The normalized ACF is defined as follows:

$$G(\tau) = \frac{\langle \delta F(t) \cdot \delta F(t+\tau) \rangle}{\langle F(t) \rangle^2}, \tag{S1}$$

where $\delta F(t)$ represents the fluctuation of fluorescence intensity in a certain time t measured on the detector, and τ, called lag-time, is the time delay relative to an earlier time point in the measurement. The fluctuations of the fluorescence intensity recorded from the detector can be written as:

$$\delta F(t) = k \int_V I_{exc}(\vec{r}) S(\vec{r}) \delta(\sigma_{abs} \Phi C((\vec{r}), t)) dV, \tag{S2}$$

with $S(\vec{r})$ as the optical transfer function of the objective-pinhole, V the volume, C the concentration of the diffusing fluorophores, σ_{abs} the cross-section for the absorption process, k the quantum efficiency for the photon detection and Φ the quantum yield for the emission. If we consider the spatial distribution of the emitted light on the detector plane:

$$W(\vec{r}) = I_{exc}(\vec{r}) S(\vec{r}) \tag{S3}$$

and the molecular brightness, defined as:

$$b = k \sigma_{abs} \Phi \tag{S4}$$

we can simplify equation S2 to:

$$\delta F(t) = \int_V W(\vec{r}) \delta(bC((\vec{r}), t)) dV. \tag{S5}$$

Considering a fluorophore with a constant brightness over time, thus, no photophysical effects, only the concentration changes are contributing to the detected fluctuation of fluorescence intensity ($\delta(bC(t)) = b\delta C(t)$). In this case, we can re-write equation S1 as:

$$G(\tau) = \frac{\iint_{VV'} W(\vec{r})W(\vec{r'}) \left\langle \delta C(\vec{r},0)\delta C(\vec{r'},\tau)) \right\rangle dV dV'}{(\int_V \delta C(\vec{r},0)W(\vec{r})dV)^2} \tag{S6}$$

with $t = 0$ for simplicity. If the fluorescently tagged molecules are freely diffusing in a three-dimensional space, the fluctuation of fluorescence given by the changes in concentrations can be described by taking advantage of the solution of the Brownian motion equation:

$$\left\langle \delta C(\vec{r},0)\delta C(\vec{r'},\tau)) \right\rangle = \langle C \rangle \frac{1}{(4\pi D\tau)^{3/2}} e^{(\vec{r}-\vec{r'})^2/4D\tau}, \tag{S7}$$

where D is the diffusion coefficient. In the confocal microscopy case, we can describe the detection plane explicitly:

$$W(\vec{r}) = I_0 e^{-2(x^2+y^2)/\omega_0^2} e^{-2z^2/z_0^2}, \tag{S8}$$

with $\vec{r} = (x, y, z)$, and ω_0 and z_0 the beam profile parameters. We can introduce the effective volume V_{eff} as:

$$V_{eff} = \frac{(\int_V W(\vec{r})dV)^2}{\int_V W(\vec{r})^2 dV}, \tag{S9}$$

which is equal to $V_{eff} = (\pi/2)^{3/2}\omega_0^2 z_0$ in the confocal case. Evaluating equation S6 with equations S7 and S9, we can explicitly write the ACF for fluorescently tagged molecules diffusing with Brownian motion in the effective volume defined by the Point Spread Function (PSF):

$$G_D(\tau) = \frac{1}{\langle C \rangle V_{eff}} \frac{1}{1+\frac{\tau}{\tau_D}} \frac{1}{\sqrt{1+\frac{\tau}{\tau_D}(\omega_0/z_0)^2}}, \tag{S10}$$

with τ_D being the diffusion time, defined as $\tau_D = \omega_0^2/4D$. The average number of molecules in the effective volume can be calculated from the average concentration as:

$$N = \langle C \rangle V_{eff}, \tag{S11}$$

while the radius of the molecule is related to its diffusion coefficient in the spherical-geometry case. In case of multiple diffusing components, the autocorrelation function has multiple (tipically two) diffusing times. In the experiments with G3BP1 under stress, some of the autocorrelation curves have been analyzed with a two components model. The first component was compatible with free protein in the dilute phase. In the case of circular scanning FCS, the fluorescence intensity is acquired by probing the sample by moving the laser beam in a circular orbit. In this case, the two parameters ω_0 and D can be decoupled by scanning the laser beam. In this case the autocorrelation

function, $G(\rho, \tau)$ is a function of both spatial shifts ρ and temporal lags τ:

$$G(\rho,\tau) = \frac{1}{N\left(1+\frac{\tau}{\tau_D}\right)\sqrt{1+\frac{\tau}{\tau_D}}} \exp\left(-\frac{\rho^2}{\omega_0^2\left(1+\frac{\tau}{\tau_D}\right)}\right). \tag{S12}$$

In the case of a laser beam scanning in circles, $\rho(\tau)$ is written as:

$$\rho(\tau) = \sqrt{R^2 + R^2 - 2R^2\cos(\gamma(\tau))} = \sqrt{2R^2\left(1-\cos\left(\frac{2\pi}{T}\tau\right)\right)}, \tag{S13}$$

where R is the radius of the scan pattern, γ is the angle over the laser beam has traveled in a time interval τ and T the time needed to scan a full circle. The parameter $\rho(\tau)$ corresponds to the distance between the two points under the angle γ. Combining Eq. S12 and S13 we obtain:

$$G(\rho,\tau) = \frac{1}{N\left(1+\frac{4D\tau}{\omega_0^2}\right)\sqrt{1+\frac{4D\tau}{\omega_0^2}}} \exp\left(-\frac{4R^2\sin^2\left(\frac{\pi}{T}\tau\right)}{\omega_0^2+4D\tau}\right). \tag{S14}$$

Since D and ω_0 now appear decoupled in the equation, they can be resolved together in a single experiment.

Whereas conventional FCS provides information about diffusion time in a given observation volume, the spatial heterogeneity of the samples is still hidden. Spot-Variation FCS is a technique where the observation volume is varied in order to probe diffusion times at different observation area sizes. For bigger enough observation volume areas, the diffusion law, $\tau_D(\omega_0^2)$ s described by:

$$\tau_D = \frac{\omega_0^2}{4D} + t_0. \tag{S15}$$

Thus, the slope of the linear regression of $\tau_D(\omega_0^2)$ is related to the diffusion coefficient, while the intercept is described by the parameter t_0. The latter is a parameter describing the "confinement" strength of the sample. In the case of Brownian motion $t_0 = 0$, in the case of hopping diffusion $t_0 > 0$, and in the case of diffusion through a meshwork t_0 <0. For smaller and smaller observation areas, the intercept converges to 0.

Supplementary Figures

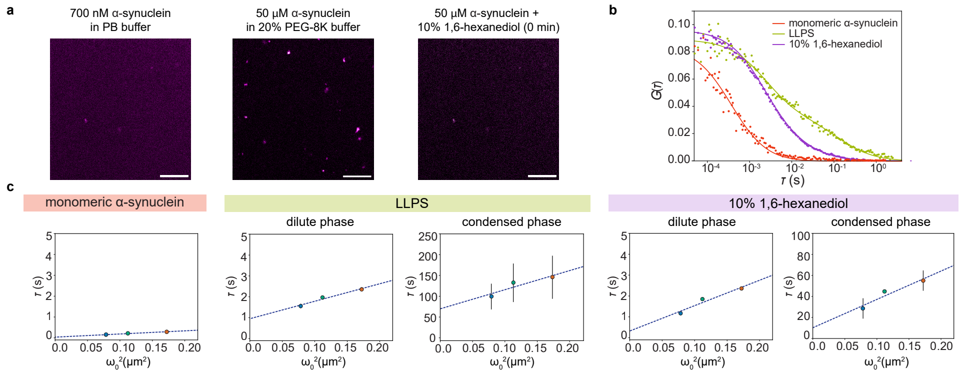

Supplementary Figure 1 Alpha-synuclein LLPS *in vitro*. a. ISM intensity-based images of 700 nM alpha-synuclein (α-syn) before LLPS (20 mM phosphate buffer (PB), *left*) and 50 µM α-syn (5% α-syn^{C141}-atto488) in LLPS buffer (20% PEG-8K, 20 mM phosphate buffer, *middle*) and immediately after the addition of 10% 1,6-hexanediol (20% PEG-8K, 20 mM PB, *right*). Scale bars 10 µm. **b.** Autocorrelation curves of α-syn in the three conditions depicted in (a). Red for α-syn in 20 mM PB, green for α-syn in LLPS buffer, and purple for dissolved α-syn condensates. While α-syn in 20 mM PB can be analyzed with one diffusing component only, α-syn after the LLPS and after the addition of 10% 1,6-hexanediol are analyzed with a two-components FCS model. A faster ($D =$ 20.0±1.5 µm^2/s for α-syn LLPS, $D =$ 18.3±0.5 µm^2/s for α-syn treated with 10% 1,6-hexanediol) and a slower ($D =$ 0.5±0.1 µm^2/s α-syn LLPS, $D =$ 0.8±0.2 µm^2/s for α-syn treated with 10% 1,6-hexanediol) diffusing components have been detected. **c.** Diffusion law plots $\tau_D(\omega_0^2)$ for the three conditions of α-syn condensation. The faster diffusing component represents the dilute phase, having an intercept t_0 close to zero (0.9 $\pm$ 0.1 ms for α-syn LLPS, 0.3 $\pm$ 0.3 ms for 10% 1,6-hexanediol treatment). The slower component represents the condensed phase, having an intercept bigger than zero (70 $\pm$ 20 ms for α-syn in LLPS, and some partial not-dissolved smaller condensates (t_0 = 13 $\pm$ 2 ms) for α-syn with %10 1,6-hexanediol).

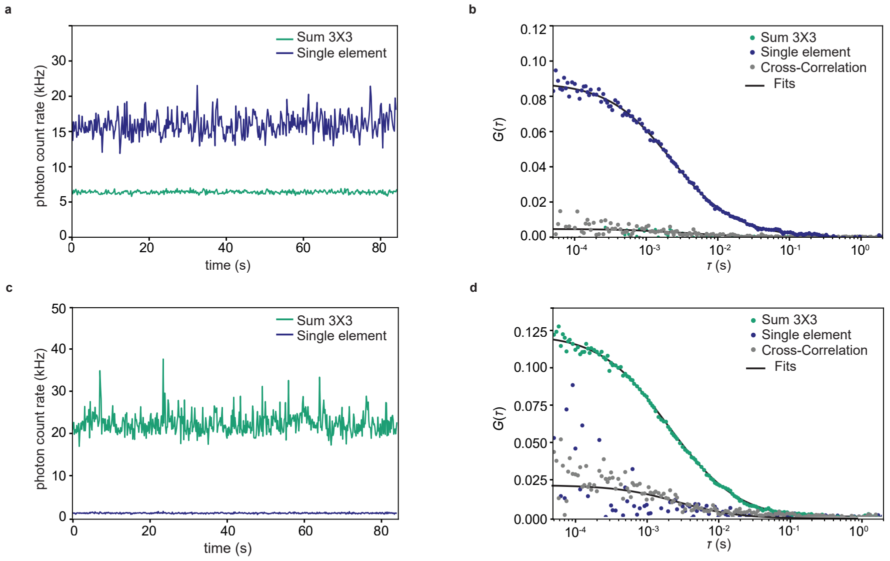

Supplementary Figure 2 Fluorescence Cross-Correlation experiment with red or yellow-green fluorescent beads only. a. Intensity time trace measured by the two detector arms for a sample of only red fluorescent beads. **b.** Dual-color Fluorescence Cross-Correlation Spectroscopy measurement of red fluorescent nanospheres in water. There is no fluorescence signal from the sample on the SPAD array detector equipped with the green filter; thus, the autocorrelation is null. On the other hand, the single-element detector, equipped with the red filter can detect the bead's signal; thus, an autocorrelation curve is calculated. As expected, the cross-correlation between the two is low. **c.** Intensity time trace measured by the two detector arms for a sample of only yellow-green fluorescent beads. **d** Dual-color Fluorescence Cross-Correlation Spectroscopy measurement of yellow-green fluorescent nanospheres in water. There is no fluorescence signal from the sample on the single-element detector equipped with the red filter; thus, the autocorrelation is null. On the other hand, the SPAD array detector, equipped with the green filter, can detect the bead's signal; thus, an autocorrelation curve is calculated. As expected, the cross-correlation between the two is low.

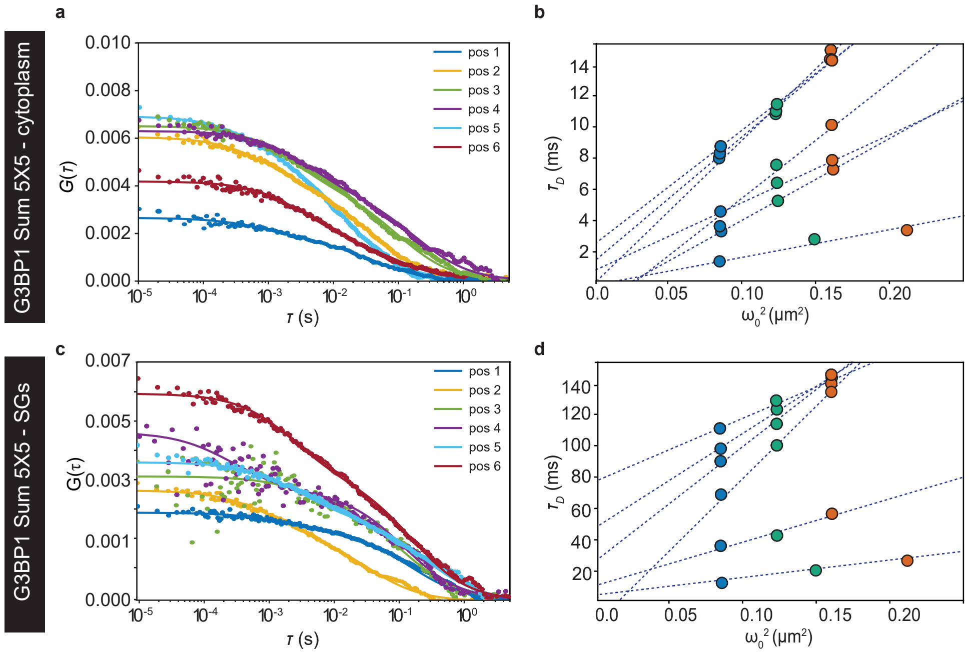

Supplementary Figure 3 FCS measurements at different positions in living cells. a. Sum 5×5 autocorrelation curve for G3BP1 measured in the cell cytoplasm before the oxidative stress in 6 different positions. **b.** Diffusion law plots ($\tau_D(\omega_0^2)$) of 6 different svFCS measurements before the stress for G3BP1. **c.** Sum 5×5 autocorrelation curve for G3BP1 measured in the cells under oxidative stress during the formation of SGs in 6 different positions. **d.** Diffusion law plots ($\tau_D(\omega_0^2)$) of 6 different svFCS measurements in SGs for G3BP1.

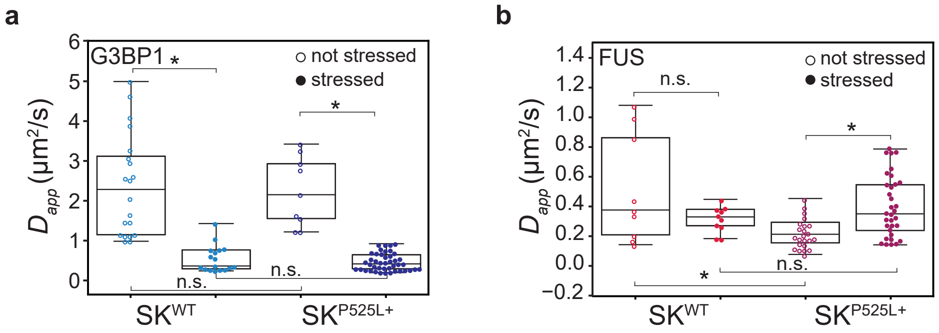

Supplementary Figure 4 G3BP1 and FUS diffusion coefficients. a. Box-plots of the apparent diffusion coefficients of G3BP1 protein before the stress, in the cytosolic phase (empty symbols), and after the stress, in the condensate phase (filled symbols), in the WT (left, n = 20 cytosolic phase and n = 17 condensate phase) and in the mutated condition (right, n = 9 cytosolic phase and n = 43 condensate phase). The horizontal line in each box represents the median, the edges are the 25th and 75th percentile, and the vertical line extends to the minimum and maximum data points. T-test confirms a change in the diffusion coefficient before the stress and inside SGs (Welch's t-test confidence level = 0.95, p-values = $3e^{-6}$ for SK-N-BE FUS WT and $3e^{-4}$ for SK-N-BE^{P525L+}), while once G3BP1 is internalized in SGs there is no significant difference in diffusion (Welch's t-test confidence level = 0.95, p-value = 0.4). **b.** Box-plots of the apparent diffusion coefficients of FUS protein before the stress, in the cytosolic phase (empty symbols), and after the stress, in the condensate phase (filled symbols), in the WT (left, n = 25 cytosolic phase and n = 17 condensate phase), and in the mutated condition (right, n = 10 cytosolic phase and n = 33 condensate phase). The horizontal line in each box represents the median, the edges are the 25th and 75th percentile, and the vertical line extends to the minimum and maximum data points. T-test confirms a change in the diffusion coefficient before the stress and inside SGs only in the mutated case (Welch's t-test confidence level = 0.95, p-values = 0.1 for SK-N-BE FUS WT and $2e^{-3}$ for SK-N-BE^{P525L+}), while before the stress we found a difference in diffusion coefficient between the WT and the mutated protein (Welch's t-test confidence level = 0.95, p-value = 0.03).

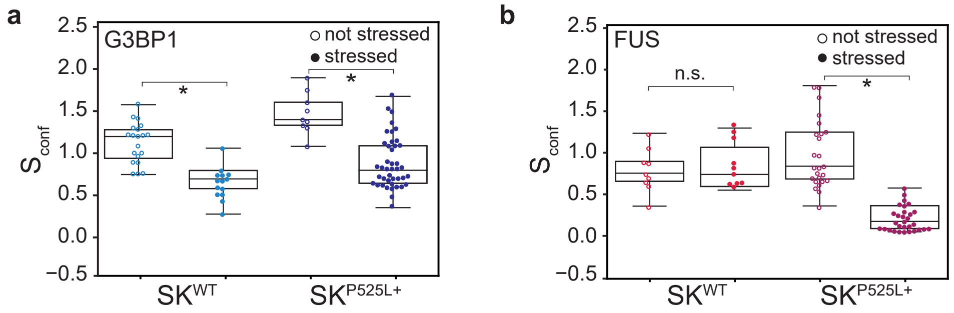

Supplementary Figure 5 G3BP1 and FUS confinement strengths. a. Box-plots of the confinement strength of G3BP1 protein before the stress, in the cytosolic phase (empty symbols), and after the stress, in the condensate phase (filled symbols), in the WT (left, n = 20 cytosolic phase and n = 17 condensate phase) and in the mutated condition (right, n = 9 cytosolic phase and n = 43 condensate phase). The horizontal line in each box represents the median, the edges are the 25th and 75th percentile, and the vertical line extends to the minimum and maximum data points. T-test confirms a change in the type of mobility before the stress and inside SGs (Welch's t-test confidence level = 0.95, p-values = $2e^{-8}$ for SK-N-BE FUS WT and $2e^{-5}$ for SK-N-BE^{P525L+}). **b.** Box-plots of the confinement strength of FUS protein before the stress, in the cytosolic phase (empty symbols), and after the stress, in the condensate phase (filled symbols), in the WT (left, n = 25 cytosolic phase and n = 17 condensate phase), and in the mutated condition (right, n = 10 cytosolic phase and n = 33 condensate phase). The horizontal line in each box represents the median, the edges are the 25th and 75th percentile, and the vertical line extends to the minimum and maximum data points. T-test confirms a change in the type of mobility before the stress and inside SGs only for the mutated cells (Welch's t-test confidence level = 0.95, p-values = 0.8 for SK-N-BE FUS WT and $4e^{-10}$ for SK-N-BE^{P525L+}).

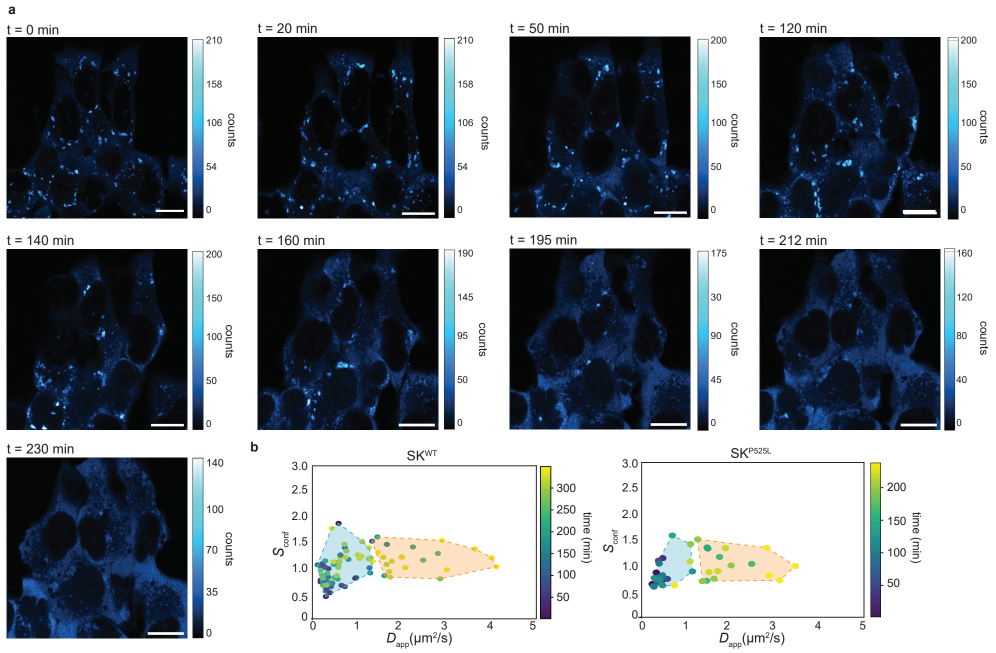

Supplementary Figure 6 Spot-Variation FCS to study stress granule recovery. a. Time-lapse ISM imaging of a typical experiment of stress recovery. SK-N-BE cells pathologically mutated, expressing G3BP1-gfp and FUS-RFP (here, only G3BP1 is shown) during stress granule recovery. Once the sodium-arsenite is removed, the stress granules dissolve. Scale bars 10 µm. **b.** Confinement strength versus diffusion coefficient for G3BP1-GFP in both WT (left) and mutated (right) cell lines. The color code represents the measurement time for each data point. Over time both the confinement strength and the diffusion coefficient retrieve values comparable to the ones before the stress. The data are clusterized based on k-mean algorithm.

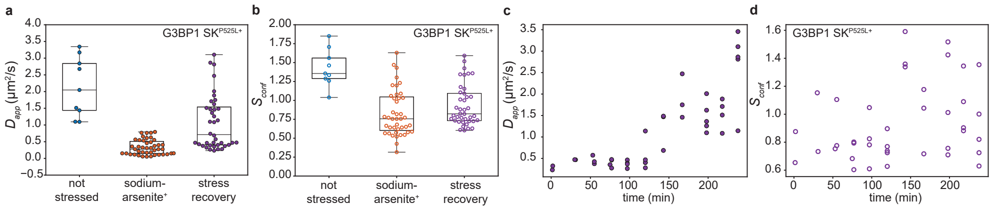

Supplementary Figure 7 Diffusion and confinement strength of G3BP1 during stress recovery. a. Box-plots of the apparent diffusion coefficients of G3BP1 protein in SK-N-BE^{P525L+} cells before stress ($n = 9$), after stress, inside the condensates ($n = 43$), and during stress recovery ($n = 41$). The horizontal line in each box represents the median, the edges are the 25th and 75th percentile, and the vertical line extends to the minimum and maximum data points. One single recovery experiment is shown. Data are acquired for 5 hours in total. **b.** Box-plots of the confinement strength of G3BP1 protein in SK-N-BE^{WT} cells before stress, after stress, inside condensates, and during the stress recovery. The horizontal line in each box represents the median, the edges are the 25th and 75th percentile, and the vertical line extends to the minimum and maximum data points. The sample sizes are the same as in (a). One single recovery experiment is shown. Data are acquired for 5 hours in total. **c.** Apparent diffusion coefficient over-time during the stress recovery experiment of G3BP1 protein in SK-N-BE^{P525L+}. One single recovery experiment is shown. Data are acquired for 5 hours. **d.** Confinement strength over time during the stress recovery experiment of G3BP1 protein in SK-N-BE^{P525L+}. One single recovery experiment is shown. Data are acquired for 5 hours.

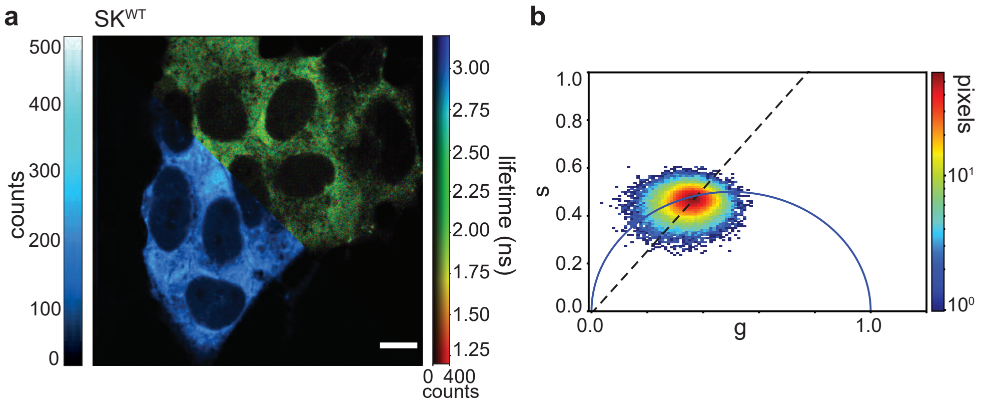

Supplementary Figure 8 FLISM and phasor analysis of SK-N-BE wt cells before oxidative stress. a. SK-N-BE WT cell expressing G3BP1-eGFP before the sodium arsenite treatment. Bottom-left ISM reconstructed intensity-based image, top-right ISM reconstructed fluorescence lifetime-based image. Scale bar 10 µm. **b.** Pixel intensity thresholded phasor plots. Number of pixels versus the polar coordinate (10% thresholds) of the image shown in (a).

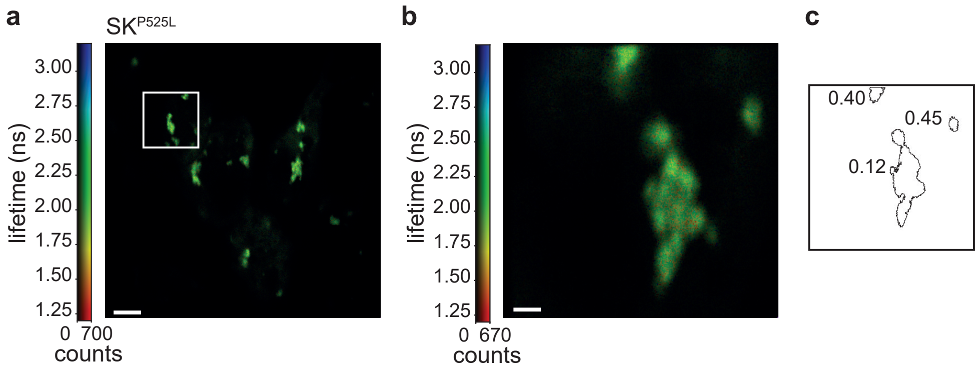

Supplementary Figure 9 Segmentation-based SG shape analysis. a. ISM reconstructed fluorescence lifetime-based image of SK-N-BE FUS^{P525L+} cells expressing G3BP1-eGFP under oxidative stress. Scale bar 10 µm. **b.** ISM reconstructed fluorescence lifetime-based image of the zoomed area in (a) in the white region-of-interest. Scale bar 1 µm. **c.** Outlines of the segmented SGs. The numbers correspond to the circularity of the segmented SG.

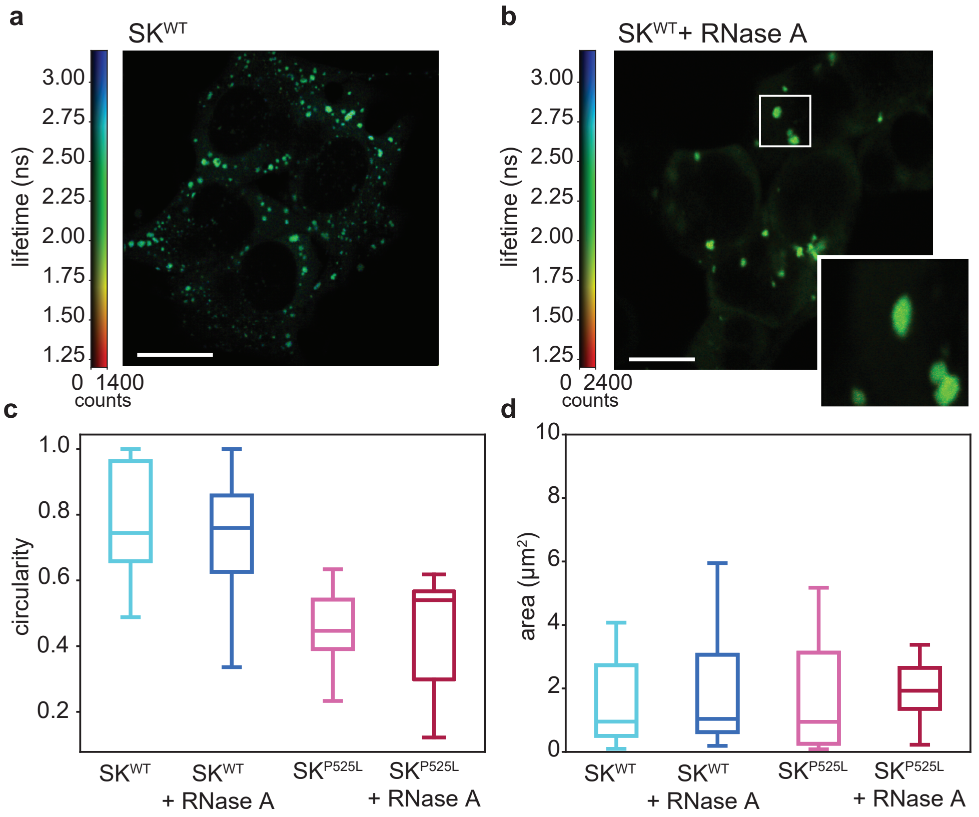

Supplementary Figure 10 RNase treatment on not permeabilized cells. a. ISM reconstructed fluorescence lifetime-based image of not permeabilized SK-N-BE WT cells expressing G3BP1-eGFP under oxidative stress before RNAseA treatment. Scale bar 10 µm. **b.** ISM reconstructed fluorescence lifetime-based image of not permeabilized SK-N-BE WT cells expressing G3BP1-eGFP under oxidative stress during the RNAseA treatment. Scale bar 10 µm. Bottom-right zoomed region in the white box. **c.** Boxplots of the circularity of the segmented SGs in SK-N-BE WT cells (left, n = 18 before RNaseA n = 35 during RNaseA, one independent experiment) and in mutated cells (right, n = 10 before RNaseA n = 6 during RNaseA, one independent experiment). The horizontal line in each box represents the median, the edges are the 25th and 75th percentile, and the vertical line extends to the minimum and maximum data points. **d.** Boxplots of the circularity of the segmented SGs in SK-N-BE WT cells (left) and in mutated cells (right). The horizontal line in each box represents the median, the edges are the 25th and 75th percentile, and the vertical line extends to the minimum and maximum data points. The sample size is the same as in (c).

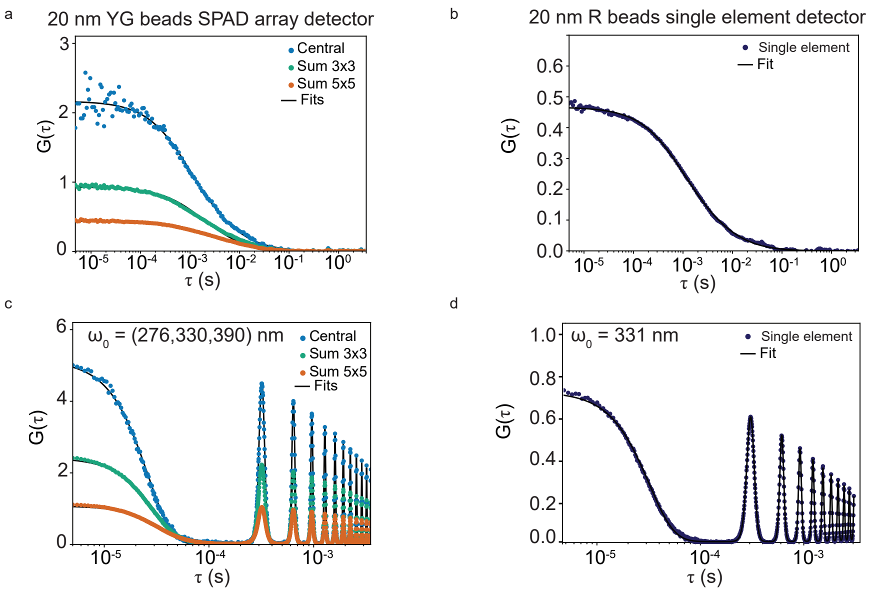

Supplementary Figure 11 Calibration of the detection volumes for FCCS. a. Example of a single-point FCS measurement of 20 nm YG fluorescent beads with the SPAD array detector. In this case, with a single measurement, we are able to retrieve the autocorrelation function for three different observation volumes (central, Sum 3×3 and Sum 5×5), thanks to the spatial characteristic of the SPAD array detector. The measured diffusion coefficient is $14.7 \pm 0.2\ \mu m^2\,s^{-1}$. **b.** Example of a single-point FCS measurement of 20 nm red fluorescent beads with the single-element SPAD detector. The measured diffusion coefficient $17.4 \pm 0.4\ \mu m^2\,s^{-1}$. **c.** Example of a circular FCS scanning measurement of 20 nm YG fluorescent beads with the SPAD array detector. The measured volume for this measurement is 276 nm for the central channel, 330 nm for the sum of the inner 3×3 channels, and 390 nm for the sum of all 25 channels. **d.** Example of a circular FCS scanning measurement of 20 nm red fluorescent beads with the single element SPAD detector. The measured volume is 331 nm, which well corresponds to the detection volume obtained for the inner 3×3 channels of the SPAD array detector.

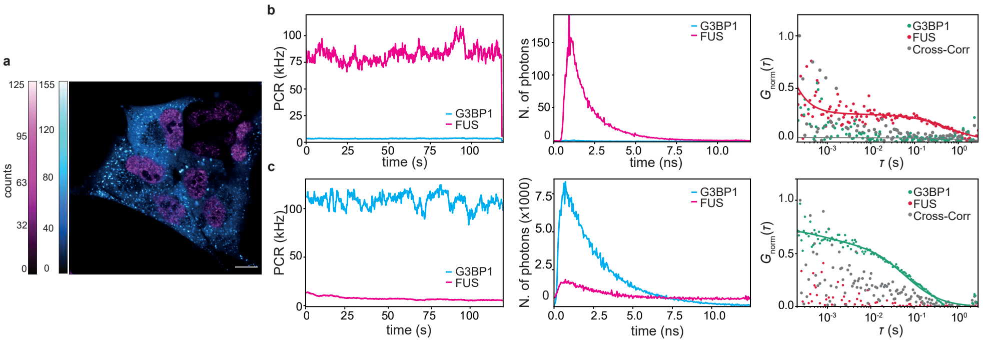

Supplementary Figure 12 FCCS on SK-N-BE WT cells under oxidative stress. a. Intensity-based ISM image of a SK-N-BE WT cell expressing G3BP1-gfp (cytoplasm, in cyan) and FUS (nucleus, magenta) after the oxidative stress. Scale bar 10 µm. **b.** FCCS experiment in the nuclei of the cells. Intensity over time (left) of G3BP1-eGFP and FUS-rfp, time decay histograms (center) for G3BP1 (cyan) and FUS (magenta) and autocorrelations and cross-correlation curves (right) (grey) calculated from the same data in the nuclei. **c.** FCCS experiment in the cytoplasm of the cells. Intensity over time (left) of G3BP1-eGFP and FUS-rfp, time decay histograms (center) for G3BP1 (cyan) and FUS (magenta) and autocorrelations and cross-correlation curves (right) (grey) calculated from the same data in the cytoplasm.

Supplementary Table 1 Characteristics of the two detectors used in the study. (*) For all experiments of this work we used 50 ns hold-off.

	Single-element SPAD	SPAD array detector
Dead time	77 ns	20, 25, 50*, 100 ns
Dark count rate	< 100 Hz	> 70 and < 1700 Hz
Fill factor	N.A.	$\sim 57{,}5$ %
Pixel size	50 μm	50 μm
Pixel pitch	N.A.	75 μm
