# Supplementary material for: Single-photon microscopy to study biomolecular condensates
Source: Nat Commun. 2023 Dec 12;14:8224. doi: 10.1038/s41467-023-43969-7 (PMC10716487; doi:10.1038/s41467-023-43969-7)
Supplement: Supplementary file 3 — Reporting Summary [file 41467_2023_43969_MOESM3_ESM.pdf]

nature portfolio

Corresponding author(s): Giuseppe Vicidomini

Last updated by author(s): Nov 17, 2023

Reporting Summary

Nature Portfolio wishes to improve the reproducibility of the work that we publish. This form provides structure for consistency and transparency in reporting. For further information on Nature Portfolio policies, see our Editorial Policies and the Editorial Policy Checklist.

Statistics

For all statistical analyses, confirm that the following items are present in the figure legend, table legend, main text, or Methods section.

n/a	Confirmed	
☐	☒	The exact sample size (*n*) for each experimental group/condition, given as a discrete number and unit of measurement
☐	☒	A statement on whether measurements were taken from distinct samples or whether the same sample was measured repeatedly
☐	☒	The statistical test(s) used AND whether they are one- or two-sided<br>*Only common tests should be described solely by name; describe more complex techniques in the Methods section.*
☒	☐	A description of all covariates tested
☒	☐	A description of any assumptions or corrections, such as tests of normality and adjustment for multiple comparisons
☐	☒	A full description of the statistical parameters including central tendency (e.g. means) or other basic estimates (e.g. regression coefficient) AND variation (e.g. standard deviation) or associated estimates of uncertainty (e.g. confidence intervals)
☐	☒	For null hypothesis testing, the test statistic (e.g. *F*, *t*, *r*) with confidence intervals, effect sizes, degrees of freedom and *P* value noted<br>*Give P values as exact values whenever suitable.*
☒	☐	For Bayesian analysis, information on the choice of priors and Markov chain Monte Carlo settings
☒	☐	For hierarchical and complex designs, identification of the appropriate level for tests and full reporting of outcomes
☐	☒	Estimates of effect sizes (e.g. Cohen's *d*, Pearson's *r*), indicating how they were calculated

Our web collection on statistics for biologists contains articles on many of the points above.

Software and code

Policy information about availability of computer code

Data collection	BrightEyes-TTM available through our GitHub repository https://github.com/VicidominiLab/BrightEyes-TTM<br>Python 3.0<br>Labview 2019
Data analysis	Python 3.0<br>ImageJ 1.53<br>FLIMJ plugin (ImageJ) 1.1.0<br>BrightEyes-TTM available through our GitHub repository (https://github.com/VicidominiLab/BrightEyes-TTM)<br>We provide a new package for the analysis on the intensity-based dataset, which is in a new folder of the BrightEyes GitHub repository (https://github.com/VicidominiLab/BrightEyes-TTM/tree/v2.0/scriptsFLFS).

For manuscripts utilizing custom algorithms or software that are central to the research but not yet described in published literature, software must be made available to editors and reviewers. We strongly encourage code deposition in a community repository (e.g. GitHub). See the Nature Portfolio guidelines for submitting code & software for further information.

Data

Policy information about availability of data

All manuscripts must include a data availability statement. This statement should provide the following information, where applicable:

- Accession codes, unique identifiers, or web links for publicly available datasets
- A description of any restrictions on data availability
- For clinical datasets or third party data, please ensure that the statement adheres to our policy

As keen proponents of open science and open data, we will make available the raw time-tagged data for the test-dataset (shown in Figure 2), which supports the findings of this study, publicly available at Zenodo (https://doi.org/10.5281/zenodo.10046694).
Full build instructions and analysis instructions for the BrightEyes-TTM are available through our GitHub repository https://github.com/VicidominiLab/BrightEyes-TTM.

The cell experiments data are available under restricted access for their large amount, full access can be obtained by request to the corresponding author. Source data are provided as a Source Data file.

Research involving human participants, their data, or biological material

Policy information about studies with human participants or human data. See also policy information about sex, gender (identity/presentation), and sexual orientation and race, ethnicity and racism.

Reporting on sex and gender	N/A
Reporting on race, ethnicity, or other socially relevant groupings	N/A
Population characteristics	N/A
Recruitment	N/A
Ethics oversight	N/A

Note that full information on the approval of the study protocol must also be provided in the manuscript.

Field-specific reporting

Please select the one below that is the best fit for your research. If you are not sure, read the appropriate sections before making your selection.

[x] Life sciences [ ] Behavioural & social sciences [ ] Ecological, evolutionary & environmental sciences

For a reference copy of the document with all sections, see nature.com/documents/nr-reporting-summary-flat.pdf

Life sciences study design

All studies must disclose on these points even when the disclosure is negative.

Sample size	Regarding the imaging experiments, in particular the FLISM measurements, we consider as sample size, the number of pixels for each field-of-view acquired were calculated based on the Nyquist sampling condition while the exposure times were decided based on the intensity flux in order to minimize photo-damage and avoid saturation. Regarding the fluorescence spectroscopy experiments, data were acquired long enough to assure enough intensity signal in order to have clear autocorrelation functions. Sample sizes and independent measurements are been reported in the manuscript.
Data exclusions	For the FFS experiments, the individual correlation curves were visually inspected and all curves without artifacts were averaged. The correlation curves with clear artifacts were excluded.
Replication	Regarding the test-measurement of fluorescent beads, data were acquired for 100 s, divided in chunks of 10 s each and averaged before the analysis. Regarding the FLFS measurements in living cells, the data were acquired for all conditions (a part for FUS protein WT where only 2 independent experiment have been performed) during 3 independent experiments where multiple cells have been tested. More than 10 points are available for all measurements. All independent experiments performed as replicates were successful.
Randomization	The methods described in the paper are not depending on randomization of the samples. This is not relevant for all the method in the paper because they do not depend on the statistical variation of the sample. For the cell experiments, cells were randomly chosen for the measurements, as well as multiple random positions within the cells.<br>We did 3 independent measurements for each condition to avoid biases in the statistical analysis.
Blinding	The cell samples were categorized depending on the mutation (wt or P525L on FUS protein). The data acquired are all analyzed in the same manner independently by the sample allocation.<br>For the cell experiments, we have done sample preparation by one experimentalist and the imaging by another experimentalist, to ensure

the reproducibility.

Reporting for specific materials, systems and methods

We require information from authors about some types of materials, experimental systems and methods used in many studies. Here, indicate whether each material, system or method listed is relevant to your study. If you are not sure if a list item applies to your research, read the appropriate section before selecting a response.

Materials & experimental systems

n/a	Involved in the study
x	Antibodies
	x Eukaryotic cell lines
x	Palaeontology and archaeology
x	Animals and other organisms
x	Clinical data
x	Dual use research of concern
x	Plants

Methods

n/a	Involved in the study
x	ChIP-seq
x	Flow cytometry
x	MRI-based neuroimaging

Eukaryotic cell lines

Policy information about cell lines and Sex and Gender in Research

Cell line source(s)	SK-N-BE(2) is a commercial cell line gently donated by the group of Irene Bozzoni.
Authentication	SK-N-BE(2) cells were not authenticated.
Mycoplasma contamination	SK-N-BE(2) cells were routinely tested negative for mycoplasma contamination by PCR assay and via DAPI nuclear staining.
Commonly misidentified lines (See ICLAC register)	SK-N-BE(2) is not included in ICLAC register as commonly misidentified line.
